# Brainstem atrophy in dementia with Lewy bodies compared with progressive supranuclear palsy and Parkinson’s disease on MRI

**DOI:** 10.1186/s12883-023-03151-4

**Published:** 2023-03-21

**Authors:** Sebastian Johannes Müller, Eya Khadhraoui, Niels Hansen, Ala Jamous, Philip Langer, Jens Wiltfang, Christian Heiner Riedel, Caroline Bouter, Christoph van Riesen, Fabian Maass, Michael Bartl, Claudia Lange, Marielle Ernst

**Affiliations:** 1 Institute of Diagnostic and Interventional Neuroradiology, University Medical Center Göttingen (UMG), Georg-August-University Göttingen, Robert-Koch-Str. 40, 37075 Göttingen, Germany; 2 Department of Psychiatry and Psychotherapy, University Medical Center Göttingen (UMG), Georg-August-University, Göttingen, Germany; 3 German Center for Neurodegenerative Diseases (DZNE), Göttingen, Germany; 4 Department of Medical Sciences, Neurosciences and Signaling Group, Institute of Biomedicine (iBiMED), University of Aveiro, Aveiro, Portugal; 5 Department of Nuclear Medicine, University Medical Center Göttingen (UMG), Georg-August-University, Göttingen, Germany; 6 Department of Neurology, University Medical Center Göttingen (UMG), Georg-August-University, Göttingen, Germany

**Keywords:** Dementia with Lewy Bodies, MRI, Atrophy, Brainstem

## Abstract

**Background:**

Although Dementia with Lewy bodies (DLB) is the second most common form of dementia in elderly patients, it remains underdiagnosed compared with Alzheimer's (AD) and Parkinson's diseases (PD). This may be explained by overlapping clinical symptoms, e.g. Parkinsonism. While current MRI research focuses primarily on atrophy patterns of the frontal and temporal lobes, we focus on brainstem characteristics of DLB. In particular, we focused on brainstem atrophy patterns distinguishing DLB from Progressive Supranuclear Palsy (PSP) and PD based as the most common differential diagnoses.

**Methods:**

We identified patients diagnosed with DLB, PD, PSP, and a control group (CTRL) in our psychiatric and neurological archives. All patients with competing diagnoses and without a high-quality T1 MPRAGE 3D dataset were excluded. We assessed atrophy patterns in all patients (1) manually and (2) using FastSurfer’s segmentation algorithm in combination with FreeSurfer’s brainstem volumetric calculations. We compared classical measurement methods and ratios with automated volumetric approaches.

**Results:**

One hundred two patients were enrolled and evaluated in this study. Patients with DLB (*n* = 37) showed on average less atrophy of the brainstem than patients with PSP (*n* = 21), but a significantly more pronounced atrophy than patients with PD (*n* = 36) and the control group (CTRL, *n* = 8). The mean measured sagittal diameters of the midbrain were 8.17 ± 1.06 mm (mean ± standard deviation) for PSP, 9.45 ± 0.95 mm for DLB, 10.37 ± 0.99 mm for PD and 10.74 ± 0.70 for CTRL. The mean measured areas of the midbrain were 81 ± 18 mm^2^ for PSP, 105 ± 17 mm^2^ for DLB, 130 ± 26 mm^2^ for PD and 135 ± 23 mm^2^ for CTRL. The mean segmented volumes of the midbrain were 5595 ± 680 mm^3^ for PSP, 6051 ± 566 mm^3^ for DLB, 6646 ± 802 mm^3^ for PD and 6882 ± 844 mm^3^ for CTRL. The calculated midbrain pons ratios did not show superiority over the absolute measurements of the midbrain for distinguishing PSP from DLB. Because of the relatively uniform atrophy throughout the brainstem, the ratios were not suitable for distinguishing DLB from PD.

**Conclusions:**

DLB patients exhibit homogenous atrophy of the brainstem and can be distinguished from patients with PSP and PD by both manual measurement methods and automated volume segmentation using absolute values or ratios.

## Introduction

Neurodegenerative diseases especially dementias show a strongly increasing prevalence in an ageing population, that reduce the quality of life of affected persons and their caregivers and create a significant economic burden [1]. Although Dementia with Lewy bodies (DLB) is the second most common form of dementia in elderly patients, it remains underdiagnosed [2]. This is probably due to the numerous clinical overlaps with other neurodegenerative diseases displaying dementia in the course, especially Alzheimer’s disease (AD) and Parkinson’s disease (PD). DLB is characterized by dementia, progressive deficits in visual spatial abilities and frontal executive functions and further clinical core features of parkinsonism, visual hallucinations and fluctuations of alertness [3]. Early and correct diagnosis is essential to ensure optimal treatment and to avoid unwanted effects from dopamine blocking agents for which DLB patients have a very high sensitivity [1]. To increase diagnostic accuracy and monitor potential therapeutic drug effects in clinical trials, there is a need to widen the research for reliable and validated biomarkers. Several biomarkers are available to improve diagnostic accuracy in DLB [4] including the atrophy patterns in Magnetic Resonance imaging (MRI). While current MRI research focuses primarily on structures of the frontal and temporal lobes [5], we would like to shed light on the atrophy patterns of the brainstem here. In particular, we are interested in how DLB fits in between the commonly studied atrophy patterns of progressive supranuclear palsy (PSP) and PD, two differential diagnoses known to affect brainstem integrity [6, 7]. Several widely used measurement methods are already available for PSP, such as the Midbrain-To-Pons-Ratio [7], the Midbrain-To-Pons-Area-Ratio [8], the Magnetic resonance parkinsonism index (MRPI) [9–11] or the MRPI 2.0 [12] but data for DLB is generally lacking. To fill in this gap, we applied automated segmentation and volumetric measurements of the brainstem in DLB, PD and PSP to assess their ability to facilitate the diagnosis and improve diagnostic confidence.

## Methods

### Participant’s population

An available high quality MRI brain scan with a 3D T1 MPRAGE dataset was the key inclusion criterion in this retrospective observational study including data from 01/01/2012 to 12/31/2021.

We analyzed patients from the psychiatric archives with clinically confirmed DLB according to the *Fourth consensus report of the DLB Consortium,* rated by an experienced psychiatrist (NH) [4].

Patients with confirmed PSP or PD were collected from the neurological archives, including all available clinical data. Diagnosis was reassessed by three movement disorder specialists (CvR, FM, MB) rating the diagnostic criteria for PSP according to Höglinger et al. [13]. PD patients were diagnosed according to UK Brain Bank and Movement Disorders Society criteria [14]. We only included subjects with PSP-RS (Richardson’s syndrome) into this analysis to increase diagnostic accuracy and reduce clinical heterogeneity in the PSP group.

The control group consisted of patients without neurological or psychiatric diseases and with a similar age and sex distribution. Exclusion criteria were large microvascular lesions (Fazekas score [15] of 2 or more) and a global cortical atrophy (GCA score [16] of 2 or more). We identified control patients using the database search of our Picture Archiving and Communication System (PACS) and confirmed them using physician letters and diagnostic findings.

### MRI analysis

3D T1 data sets were acquired with two different MRI scanners (1.5 Tesla Siemens AvantoFit and 3.0 Tesla Siemens Magnetom/PrismaFit). We included only patients with T1 MP-RAGE (Magnetization Prepared—RApid Gradient Echo) sequences. Patients who had only T1 VIBE (Volumetric interpolated breath-hold examination) sequences were excluded because of the lower contrast between white and grey matter.

All subjects were scanned in sagittal orientation with a voxel resolution of 1.0 × 1.0 × 1.0 mm (parameters for 1.5 T MP-RAGE: scan time 298 s, TR 1.700 ms, TE 2.460 ms, flip angle 8°, TI 900 ms, 3.0 T: scan time 260 s, TR 2.000 ms, TE 2.980 ms, flip angle 9°, TI 900 ms; and for 3.0 T MP-RAGE: scan time 260 s, TR 2.000 ms, TE 2.980 ms, flip angle 9°, TI 900 ms).

We retrospectively analyzed MRIs with FastSurfer/FreeSurfer and three independent raters blinded to diagnosis. The raters were radiologists with 5 years (rater 1, EK), 6 years (rater 2, AJ) and 4 years (rater 3, SM) of experience in neuroradiologic MR imaging of dementia.

### Automated volumetric MRI analysis

**Fig. 1:**
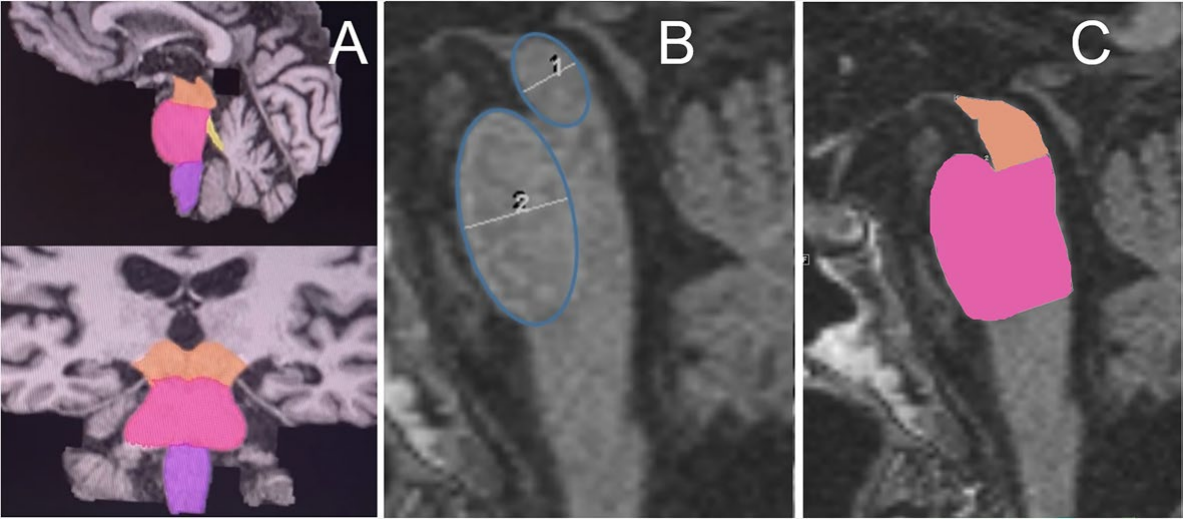
Illustration of automated (**A**) and manual (**B**,** C**) measurements. Legend: **A** FastSurfer’s segmentation with FreeSurfer’s brainstem script of midbrain (orange), pons (pink), medulla oblongata (purple) and superior cerebellar peduncle (yellow), visualized using 3D slicer. **B** Drawn ellipsoids of midbrain (1) and pons (2), for the measurements of the diameters. **C** Manual area segmentation on sagittal plane of midbrain (orange) and pons (pink)

In the first step, we used the 3D Slicer Software (Version 4.10.2, https://www.slicer.org/) to convert the DICOM (Digital Imaging and Communications in Medicine) file format to the NIFTI (Neuroimaging Informatics Technology Initiative) file format. For segmentation, FastSurfer [17] was used (Version commit dabf1e02e6253cac8bd3d641958b01e5348ea0e7, https://github.com/Deep-MI/FastSurfer/commit/dabf1e02e6253cac8bd3d641958b01e5348ea0e7) with the procedure call: run_FastSurfer.sh –fs_license $FREESURFER _HOME/license.txt –sd $out_path –sid $filename –t1 $f/$filename.nii –parallel –threads 24 –batch 64 –order 3 –vol_segstats. Surface statistics were obtained via FMRIB Software Library v6.0 (FSL 6.0), Version 6.0.4, from https://fsl.fmrib.ox.ac.uk/fsl/fslwiki/FslInstallation recon_surf). Used graphic card was GPU nVidia GV100, Driver 455.45.01, CUDA Version 11.1. Operating sytem was Ubuntu 18.04.5 LTS. Data from each patient’s stats folder was stored in a separate Excel-file. After visual control of the FastSurfer segmentation, we executed FreeSurfer’s brainstem script [18, 19]. For each segmentation, the volumes of medulla oblongata, pons, superior cerebellar peduncle, midbrain and the whole brainstem were analyzed and controlled visually for correctness. An example of the segmented areas is shown in Fig. 1A. The average computation time for the entire procedure was 20 min (8 min for the segmentation and 12 min for the brainstem script).

### Manual metric MRI analysis

Each rater separately and without knowledge of patient information measured the diameter and area of the midbrain and pons according to the calculation of the midbrain-to-pons-ratio (using elliptical regions of interest) [7], and the midbrain-to-pons-area-ratio [8], respectively. The software used was GE Centricity RA1000 (GE Healthcare, Chicago, Illinois, USA). An example of the measurements is shown in Fig. 1B, C.

### Statistical analysis

We used the Statistica program, version 13 (TIBCO Software Inc., Palo Alto, CALIFORNIA, USA). The significance level was set at *P* < 0.05. Interrater agreement was assessed by calculating the intraclass correlation coefficient (ICC, type: two-way, absolute agreement) for the measurements [20]. The latter was calculated using the libraries of r Version 4 (https://www.r-project.org/): irr, readxl, lpSolve and psych. Values were interpreted according to Koo and Li [21]. An ANOVA (analysis of variance) posthoc analysis was done with Tukey’s Test [22] in r.

Receiver-operating-characteristics (ROC) with area-under-curve calculations and Youden’s J analyses were used to estimate the optimal thresholds to discriminate the groups. We used the t-test to detect significant differences in the distribution or median.

Corrections for multiple comparisons were performed with the Bonferroni method [23].

## Results

### Participants

**Fig. 2:**
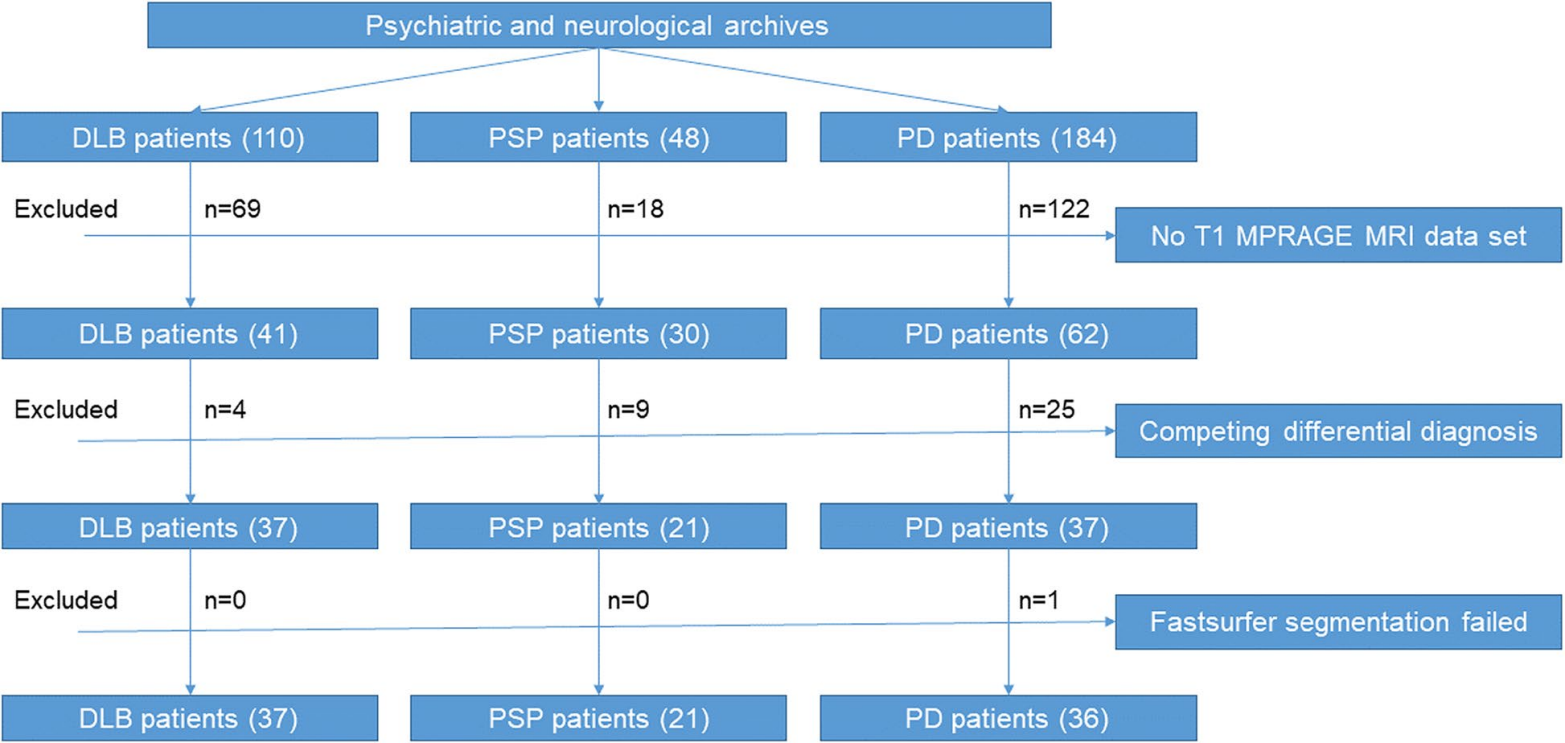
Flowchart of included patients. Legend: DLB—Dementia with Lewy bodies; PSP – progressive supranuclear palsy; PD – Parkinson’s disease

**Fig. 3:**
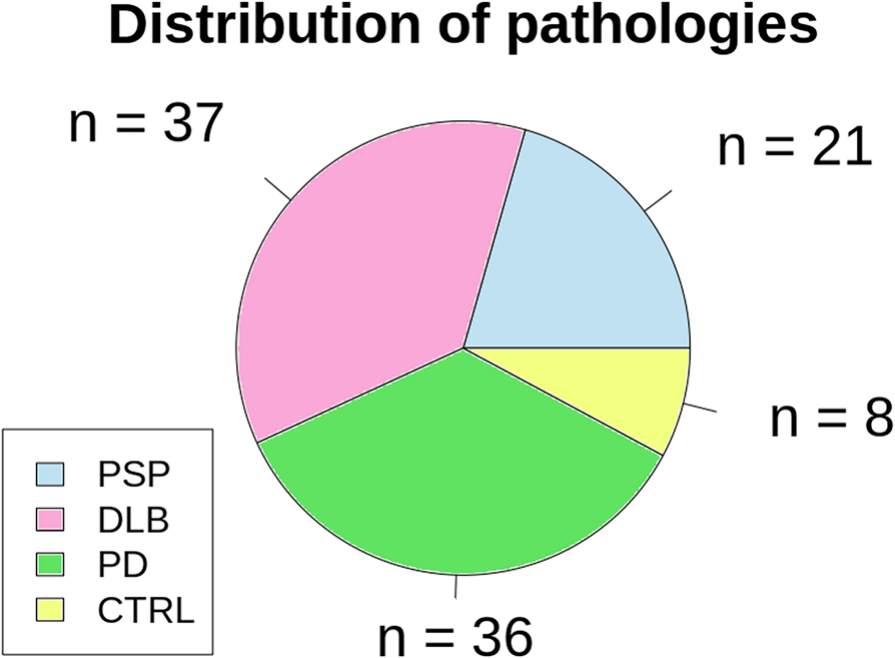
Pie chart of diseases and controls. Legend: PSP—progressive supranuclear palsy; DLB—dementia with Lewy bodies; PD—Parkinson’s disease; CTRL – control group

We were able to confirm the diagnosis of DLB according to the *Fourth consensus report of the DLB Consortium* [4] in a group of 110 patients with DLB from our psychiatric archives. In our neurological archives, we found 48 patients with PSP and 184 patients with PD. We excluded 69 patients with DLB, 18 patients with PSP, and 122 patients with PD due to an insufficient MRI (missing T1 MPRAGE or movement artifacts). Four DLB, Nine PSP and 26 PD patients were excluded because of a competing differential diagnosis. Finally, 37 patients with DLB, 21 patients with PSP, 36 patients with PD and 8 control patients (3 women, 67.2 ± 9.3 years) were included. A flowchart of included patients and a pie chart of distributed diseases are shown in Figs. 2 and 3, respectively.

### Clinical data

The mean disease duration at the time of MR scan was 2.9 ± 2.4 years in patients with PSP (*n* = 21, 7 females, 72.1 ± 6.5 year), 2.8 ± 2.6 years in patients with DLB (*n* = 37, 18 women, mean age ± standard deviation: 75.4 ± 7.7 years) and 2.6 ± 2.4 years in patients with PD (*n* = 36, 14 women, 68.3 ± 10.2 years).

In patients with DLB, the last documented Mini–Mental State Examination (MMSE) before MRI was 21.7 ± 5.8 points. In PD patients, mean Hoehn and Yahr score [24] was 2.3 ± 1.0. A subgroup of six patients (mean age 77.1 ± 6.9 years) suffered from Parkinson’s disease dementia (PDD).

### Automated volumetric MRI analysis

The mean segmented volumes of the midbrain were 5595 ± 680 mm^3^ for PSP, 6051 ± 566 mm^3^ for DLB, 6646 ± 802 mm^3^ for PD, and 6882 ± 844 mm^3^ for the control group. Volumes differed significantly between the PSP and DLB group, t-test, *p* < 0.01, the PSP and PD group, t-test, *p* < 0.001, and between DLB and PD, t-test, *p* < 0.003. There were no significant differences between PD and the control group (t-test, *p* > 0.5).

**Fig. 4:**
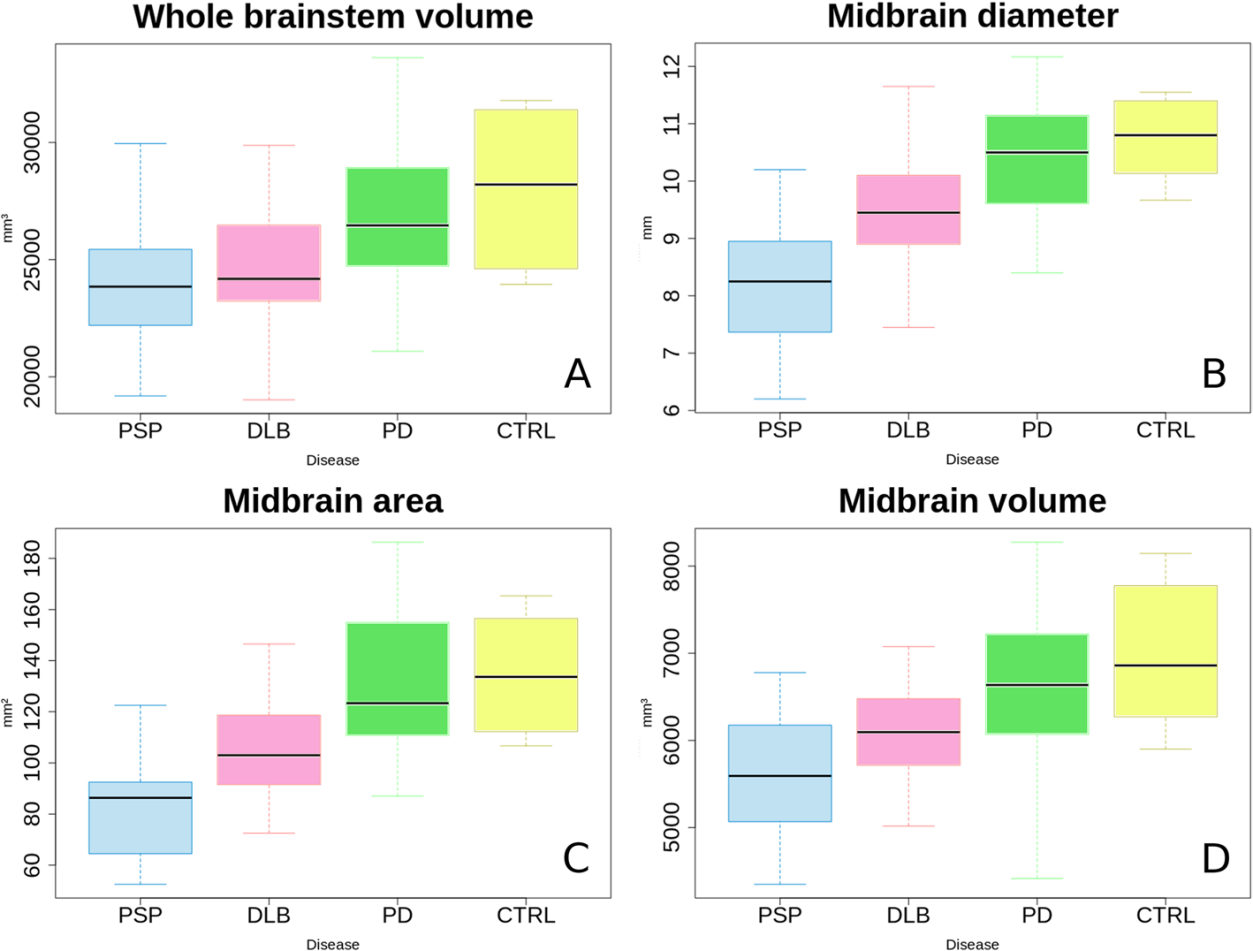
Box charts of the measured and segmented absolute values. Legend: PSP—progressive supranuclear palsy; DLB—dementia with Lewy bodies; PD—Parkinson’s disease; CTRL – control group

The mean values of all volumes are shown in Table 1. A boxplot of the absolute volumes of the midbrain and whole brainstem is shown in Fig. 4A and D.

**Table 1:** Manual and volumetric analysis of selected structures

Structure	PSP (*n* = 21)		DLB (*n* = 37)		PD (*n* = 36)		Control (*n* = 8)	
	**Mean**	**SD**	**Mean**	**SD**	**Mean**	**SD**	**Mean**	**SD**
Diameter midbrain^a^ (in mm)	8.17	1.06	9.45	0.95	10.37	0.99	10.74	0.70
Diameter pons^a^ (in mm)	16.81	1.50	16.69	1.31	17.41	1.18	17.94	0.96
Diameter ratio in %^a^ midbrain/pons	49	58	57	7	60	5	60	6
Area midbrain^a^ (in mm^2^)	81	18	105	17	130	26	135	23
Area pons^a^ (in mm^2^)	449	58	505	56	534	50	546	51
Area ratio in %^a^ midbrain/pons (in per cent)	16	3	21	3	25	5	25	5
Volume midbrain (in mm^3^)	5595	680	6051	566	6646	802	6882	844
Volume pons (in mm^3^)	13,678	1726	13,797	1699	15,161	1845	15,787	1987
Volume ratio in %^a^ midbrain/pons	41	3	44	3	44	3	44	2
Volume SCP (in mm^3^)	245	51	256	45	280	53	282	67
Volume ratio in %^a^ (midbrain + SCP)/pons	43	3	46	3	46	3	46	2
Volume medulla oblongata (in mm^3^)	4354	550	4397	448	4790	557	4982	541
Volume whole brainstem (in mm^3^)	23,827	2814	24,501	2624	26,908	3065	28,020	3322

*PSP* progressive supranuclear palsy, *DLB* Dementia with Lewy Bodies, *PD* Parkinson’s disease, *SCP* superior cerebellar peduncle

^a^Mean value of three raters

In the subgroup of patients with PDD, the mean segmented volume of the midbrain was 6286 ± 1081 mm^3^.

### Manual measurements

Mean measured midbrain diameters were 8.17 ± 1.06 mm (mean ± standard deviation) for PSP, 9.45 ± 0.95 mm for DLB, 10.37 ± 0.99 mm for PD, and 10.74 ± 0.70 for the control group. T-tests demonstrated significant differences between all groups, with the exception of the comparison of PD and the control group with *p* = 0.18.

The mean measured areas of the midbrain were 81 ± 18 mm^2^ for PSP, 105 ± 17 mm^2^ for DLB, 130 ± 26 mm^2^ for PD, and 135 ± 23 mm^2^ for CTRL. T-test demonstrated similar results as above. The *p*-values of pairwise t-tests are shown in Table 2. To confirm the results, we performed an additional analysis of variance (ANOVA) post hoc, which is presented in Table 3.

**Table 2:** Results of paired t-Test’s for measured diameters, areas and volumes

		**DLB**	**PD**	**CTRL**
**PSP**	p(DIA) p(AREA) p(VOL)	< 0.0001^b^, < 0.00001^b^, < 0.008^b^	< 0.000001^b^, < 0.000001^b^, < 0.00001^b^,	< 0.000001^b^, < 0.000001^b^, < 0.0001^b^
**DLB**	p(DIA) p(AREA) p(VOL)	-	< 0.0002^b^, < 0.00001^b^, < 0.004^b^	< 0.0002^b^, < 0.001^b^, < 0.005^b^
**PD**	p(DIA) p(AREA) p(VOL)	< 0.0002^b^, < 0.00001^b^, < 0.004^b^	-	0.68, 0.32, 0.30

*PSP* progressive supranuclear palsy, *DLB* Dementia with Lewy Bodies, *PD* Parkinson’s disease, *CTRL* control group; p significance

^b^corrected level of significance < 0.0083 (6 pairs; Bonferroni); DIA, AREA, VOL—diameters, areas (regarding the mean measured values of three raters) and volumes

**Table 3:** Results (*p*-values) of Tukey’s test (ANOVA post hoc) for six pairwise comparisons of four groups

Group	PSP-DLB	PSP-PD	PSP-CTRL	DLB-PD	DLB-CTRL	PD-CTRL
**Dia(midbrain)**	< 0.001^b^	< 0.001^b^	< 0.001^b^	< 0.001^b^	0.005^b^	0.764
**Dia(pons)**	0.983	0.291	0.134	0.064	0.053	0.704
**Area(midbrain)**	< 0.001^b^	< 0.001^b^	< 0.001^b^	< 0.001^b^	0.003^b^	0.958
**Area(pons)**	0.684	0.014^a^	0.060	0.101	0.222	0.949
**Volume(midbrain)**	0.088	< 0.001^b^	< 0.001^b^	0.003^b^	0.005^b^	0.614
**Volume(pons)**	0.995	0.016^a^	0.027^a^	0.008^b^	0.026^a^	0.805
**Ratio(diameter)**	< 0.001^b^	< 0.001^b^	< 0.001^b^	0.206	0.53	0.998
**Ratio(area)**	< 0.001^b^	< 0.001^b^	< 0.001^b^	< 0.001^b^	0.048^a^	0.993
**Ratio(volume)**	< 0.001^b^	< 0.001^b^	0.0157^b^	0.993	0.996	0.980
**Volume(SCP)**	0.854	0.060	0.297	0.180	0.555	0.999
**Volume(brainstem)**	0.854	0.001^b^	0.004^b^	0.003^b^	0.012^a^	0.756

*SCP* superior cerebellar peduncle, *PSP* progressive supranuclear palsy, *DLB* dementia with Lewy bodies, *PD* Parkinson’s disease, *CTRL* control group

^a^significant at a significance level of 0.05; ^b^significant at a corrected level of 0.0083 (6 pairs; Bonferroni)

In the subgroup of patients with PDD, the mean measured midbrain diameter and area were 10.2 ± 8 mm and 122 ± 18 mm^2^, respectively.

Figure 4B and C demonstrate a boxplot of the measured diameters and volumes.

### Interrater reliability

The single score intraclass correlation’s (3 raters, type agreement) were ICC(A,1) = 0.84 for the diameter (good reliability) and ICC(C,1) = 0.92 for the area measurements (excellent reliability).

### Differentiating of groups

The best combined marker for the differentiation of PSP, DLB and PD using Youden’s J analyses of Area-under-curve was the measured midbrain area with an optimal threshold of 94 mm^2^ for differentiation of PSP and DLB (ROC AUC 0.83, sensitivity 86%, specificity 60%) and 117 mm^2^ for DLB vs. PD (ROC AUC 0.79, sensitivity 73%, specificity 69%).

The thresholds for the measured brainstem diameter in the sagittal plane were 8.6 mm for differentiation of PSP and DLB (ROC AUC 0.82, sensitivity 67%, specificity 84%), and 9.6 mm for DLB vs. PD (ROC AUC 0.75, sensitivity 65%, specificity 75%).

**Fig. 5:**
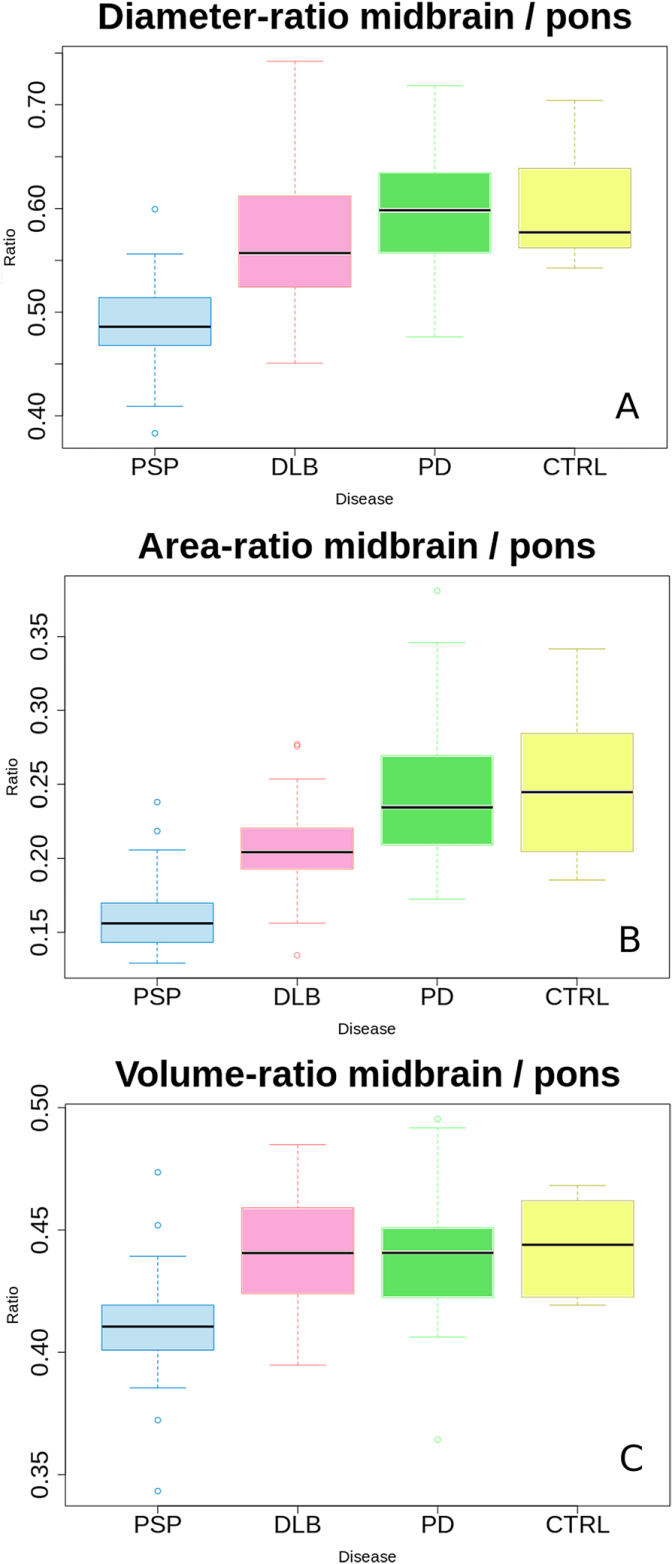
Box charts of the calculated ratios. Legend: PSP—progressive supranuclear palsy; DLB—dementia with Lewy bodies; PD—Parkinson’s disease; CTRL – control group

The diameter ratio demonstrated a sensitivity of 87% and specificity of 77% in distinguishing PSP and DLB with at a threshold of 0.524 (ROC AUC 0.86). The area ratio performed second best a sensitivity of 76% and a specificity of 88% at a threshold of 0.17 (ROC AUC 0.85). The volume ratio (midbrain:pons) performed worst with a sensitivity of 76% and specificity of 78% at a ratio of 0.422 (ROC AUC 0.80). The use of the additional volume ratio (midbrain + superior cerebellar peduncle:pons) minimally improved sensitivity and specificity (ROC AUC 0.81). Because of the relatively uniform atrophy of the entire brainstem, the ratios were inappropriate for distinguishing DLB from PD. Figure 5 demonstrates a boxplot of the calculated ratios. The mean calculated ratios in the subgroup of patients with PDD were 0.61 ± 0.06 (diameter), 0.24 ± 0.04 (area) and 0.43 ± 0.04 (volume).

Patients with DLB showed less atrophy than patients with PSP in all measured and calculated brainstem parameters, but significantly more pronounced atrophy than patients with PD and the control group.

## Discussion

Brainstem atrophy in DLB is less analyzed and poorly understood compared to other Parkinsonian disorders, though several symptoms indicate underlying brainstem pathologies [25]. Brainstem atrophy pattern are well studied in PSP and PD, including the cerebellum in PSP [26]. On the contrary, for other neurodegenerative diseases, like DLB, there are only a few studies, for example on the non-fluent primary progressive aphasias, available [27]. Therefore, we assessed brainstem parameters in DLB patients on MRI. The group of 102 patients comprised of DLB (37) and PSP (21), PD (36) and non-neurological subjects as controls. We applied state of the art segmentation algorithms and brainstem volumetric calculations by implementing the FreeSurfer brainstem script on FastSurfer segmentation, with an absolute calculation time of approx. 20 min per patient. Thus, we were able to demonstrate moderate brainstem atrophy in DLB patients, which can be classified between the pronounced atrophy in PSP and the non-significant atrophy in PD patients.

Comparison with classical brainstem measurements showed a general agreement of volumetric results with a slightly worse sensitivity and specificity. We could show that manual measurements and automated volumetric calculations can be helpful not only in the differential diagnostics of PD, PSP and Multiple System Atrophy, as already known [28], but also of DLB. Since automated categorization methods are already available for PD, PD and MSA in clinical settings [29], symptom-based interpretation of measured and volumetric results may also improve diagnostic confidence in DLB.

By showing significant differences between PSP and PD, our results are consistent with previous MRI studies, analyzing brainstem atrophy [30, 31], as well as with combined MRI and pathologic studies [32].

For future studies, it would be particularly interesting to assess longitudinal data by evaluating atrophy progression in DLB over time, as it has been demonstrated to distinguish PSP from PD based on 1-year decline in midbrain-to-pons ratio [33].

The additional use of further imaging and non-imaging biomarkers could also be helpful here. Other MRI-based clinical radiological correlations have already been demonstrated to distinguish PSP, MSA-P, and PD [34] and for PSP, early diagnostic accuracy could be improved using a combination of cerebrospinal fluid (CSF) tau ratio and brainstem atrophy [35].

Further, atrophy patterns in the frontal and temporal regions could support the diagnosis of DLB [5, 36]. Quantitative MRI measurements could expand the spectrum with newly developed high-resolution T1 or T2 mappings [37–42], by recognizing and measuring pathologic white matter changes.

## Limitations

Usage of an automated web-based MPRI calculation [43] was not possible for data protection reasons.

Another limitation is that we did not compare our groups with patients with multiple system atrophy [44], also an important but rare differential diagnosis, because we could not include enough patients.

## Conclusion

DLB patients exhibit a homogenous atrophy pattern of the brainstem and can be distinguished from patients with PSP and PD by both manual measurement methods and automated volume segmentation. The midbrain-pons-ratios are well suited to distinguish PSP and DLB, but not DLB and PD.

## Data Availability

The datasets used and analyzed in the current study are available from the corresponding author upon request. Individual patient data were not published, so no identification can be made.

## Abbreviations

ANOVA: Analysis of variance
AUC: Area under curve
cMRI: Cranial magnetic resonance imaging
CTRL: Control group
DLB: Dementia with Lewy bodies
ICC: Interclass correlation coefficient
MCI: Mild cognitive impairment
MR: Magnetic resonance
PD: Parkinson’s disease
PSP: Progressive supranuclear palsy
ROC: Receiver operating characteristics
SD: Standard deviation
MP-RAGE: Magnetization Prepared—RApid Gradient Echo
VIBE: Volumetric interpolated breath-hold examination
